# Linsitinib Decreases Thyrotropin-Induced Thyroid Hormone Synthesis by Inhibiting Crosstalk Between Thyroid-Stimulating Hormone and Insulin-Like Growth Factor 1 Receptors in Human Thyrocytes *In Vitro* and *In Vivo* in Mice

**DOI:** 10.1089/thy.2024.0393

**Published:** 2025-02-07

**Authors:** Alisa Boutin, Elena Eliseeva, Scott Templin, Bernice Marcus-Samuels, D. Eric Anderson, Marvin C. Gershengorn, Susanne Neumann

**Affiliations:** ^1^ Laboratory of Endocrinology and Receptor Biology, Bethesda, Maryland, USA.; ^2^ Advanced Mass Spectrometry Core, National Institute of Diabetes and Digestive and Kidney Diseases, National Institutes of Health, Bethesda, Maryland, USA.

**Keywords:** TSHR, IGF-1R, crosstalk, linsitinib, thyroid hormone synthetic genes

## Abstract

**Background:**

Thyrotropin receptor (TSHR) and insulin-like growth factor 1 receptor (IGF-1R) have been shown to crosstalk in primary cultures of human thyrocytes (hThyros) and Graves’ orbital fibroblasts. The phenomenon of TSHR/IGF-1R crosstalk has been largely studied in the pathogenesis of thyroid eye disease (TED) in human orbital fibroblasts. Here, we investigated the effects of inhibiting the IGF-1R-mediated contribution to crosstalk by linsitinib (Lins), a small-molecule IGF-1R kinase inhibitor, on TSH-induced regulation of thyroperoxidase (TPO) and thyroglobulin (TG) mRNAs and proteins in hThyros *in vitro,* and on TPO and TG mRNAs and free thyroxine (fT4) levels *in vivo* in mice.

**Methods:**

Steady-state levels of mRNAs of TPO and TG in hThyros *in vitro* and mouse thyroid glands were measured by RT-qPCR. Human TG (hTG) and human TPO (hTPO) proteins in human thyroid cell cultures were measured by Western blot or ELISA. Translation rates of hTG were quantified by stable isotope labeling by amino acids method (SILAC). Thyroidal mouse *Tpo* (m*Tpo*) and *Tg* (m*Tg*) mRNAs and fT4 in mice were assessed after Lins administration on 3 consecutive days followed by an intraperitoneal dose of bovine TSH (bTSH) 3 hours prior to drawing blood.

**Results:**

In primary cultures of hThyros, Lins inhibited bTSH-induced upregulation of hTPO mRNA by 61.5%, and hTPO protein was inhibited by 42.4%. There was no effect of Lins on hTG mRNA, but Lins inhibited the upregulation of secreted and cell-associated hTG protein by 50.1% and 42.2%, respectively, by inhibiting hTG mRNA translation. m*Tpo* mRNA measured in thyroid glands after treatment with Lins was reduced by 31.5%. There was no effect of Lins on m*Tg* mRNA, however, Lins decreased fT4 levels in mice under basal (endogenous mTSH levels) and bTSH-treated conditions.

**Conclusions:**

The IGF-1R antagonist Lins inhibited bTSH-stimulated hTG and hTPO protein expression in primary cultures of hThyros and fT4 levels in mice. We suggest that thyroid function studies be monitored when Lins is administered to humans, for example, if it is used to treat TED.

## Introduction

The functional effects of thyrotropin (TSH) activation of thyrotropin receptor (TSHR), a seven transmembrane-spanning receptor (7TMR), are modified by simultaneous activation of insulin-like growth factor 1 receptor (IGF-1R), a receptor tyrosine kinase (RTK), in thyrocytes,^1–3^ reviewed in.^4^ This phenomenon has been observed in nonhuman thyroid-derived cell lines^2–4^ and primary cultures of hThyros.^5^ Synergistic increases in DNA synthesis or cell cycle progression were seen in rodent cell lines and dog thyrocytes following simultaneous activation of both receptors,^4^ whereas in sheep thyrocytes, combined TSH and IGF-1 treatment was needed to stimulate thyroid function.^1^ In hThyros, TSH and IGF-1 cotreatment had additive effects on thyroid hormone synthetic gene mRNAs.^5^ TSHRs and IGF-1Rs physically interact in primary cultures of GOFs and in normal hThyros,^6^ suggesting the presence of signaling complexes containing both receptors. Interactions between TSHRs and IGF-1Rs, termed TSHR/IGF-1R crosstalk, have mainly been studied in GOFs.^7,8^ It is important to note that 7TMR/RTK crosstalk is a ubiquitous phenomenon.^9–13^

Targeting IGF-1R is a promising therapeutic strategy for thyroid eye disease (TED), as it signals in concert with TSHR. One way to study the role of IGF-1R in TSHR/IGF-1R crosstalk is to inhibit IGF-1R activation during TSHR activation by TSH. Therapeutic IGF-1R blocking antibodies, such as teprotumumab, have been used to explore IGF-1R’s role in TED.^14^

Linsitinib (Lins), a small molecule IGF-1R kinase inhibitor, has shown effectiveness in GOFs^15^ and fibrocytes,^16,17^ cells involved in the pathogenesis of TED. Recent studies in a mouse model of Graves’ disease (GD) have demonstrated that Lins can prevent the development and progression of TED.^18^ Currently, Lins is undergoing a clinical trial for TED treatment (ClinicalTrials.gov Identifier: NCT05276063).

Considering Lins’ potential importance in TED, we further studied its effects on thyroid cell function. Our findings show that Lins inhibits the TSH-induced upregulation of human thyroperoxidase (hTPO) mRNA and protein. Although Lins has no effect on human and mouse thyroglobulin (TG) mRNAs, it does inhibit the translation of hTG, reducing cell-associated and secreted hTG. Moreover, we confirmed the inhibitory effects of Lins on thyroid hormone synthesis in mice.^16^

## Materials and Methods

### Materials

Dulbecco’s modified Eagle’s media (DMEM), 1 M HEPES buffer, 100-fold penicillin-streptomycin (P/S) solution, phosphate-buffered saline (PBS), and Hank’s Balanced Salt solution were obtained from Mediatech Inc. (Manassas, VA). Fetal bovine serum (FBS) was purchased from GE Healthcare (Logan, UT). Bovine TSH (bTSH), Human Thyroglobulin ELISA kits, pharmaceutical-grade Kolliphor-ELP, and dimethylacetamide were obtained from Millipore Sigma (Burlington, MA). Lins was purchased from Selleckchem (Houston, TX). Costar 12-well Clear TC-treated Multiwell Plates were obtained from Corning Life Sciences (Corning, NY). RNeasy Mini and Micro kits were purchased from Qiagen (Germantown, MD). cDNA Archive Kit was purchased from Applied Biosystems (Foster City, CA). iTaq Universal Probe Supermix was obtained from Bio-Rad Laboratories (Hercules, CA). Trypsin, Taqman probes, NuPAGE precast Bis-Tris gels, NuPAGE MOPS sodium dodecyl sulfate (SDS) running buffer, Halt^TM^ protease and phosphatase inhibitor cocktail (100×), RIPA lysis buffer and nitrocellulose membrane sandwiches, anti-TPO rabbit polyclonal antibodies (Cat # PA5-81070, RRID: AB_2788319), DMEM for pSILAC, medium and heavy labeled lysine and arginine amino acids were purchased from ThermoFisher Scientific (Waltham, MA.) Bovine serum albumin (BSA) was purchased from MP Biomedicals (Santa Ana, CA). Odyssey blocking buffer and IRDye secondary antibodies were purchased from Li-COR Biotechnology (Lincoln, NE). Anti-TG rabbit monoclonal (Cat # ab156008, RRID: AB_2922822) and anti-β-tubulin mouse monoclonal (Cat # ab231082, RRID: AB_2922824) antibodies were obtained from Abcam (Cambridge, MA). Heparinized collection tubes were obtained from DWK Life Sciences, LLC (Millville, NJ) and Free T4 AccuBind ELISA was from Monobind Inc. (Lake Forest, CA). Isofluorane was purchased from Baxter International Inc. (Deerfield, IL).

### Animals

All experiments were approved by the NIDDK Institutional Animal Care and Use Committee (Protocol number: K019-LERB-22) and conducted in an AALAC-accredited NIH vivarium. Ten-week-old wild-type female BALB/cAnNCrl mice (*n* = 96; strain code: 555) were purchased from the Charles River Laboratories NCI Grantee Program. Mice were housed four per cage and acclimated to our vivarium for 1 week before initiating experiments at 11 weeks of age. All experiments were performed in the dark phase of the light cycle, at approximately the same time of day, beginning just after lights out. Mice were housed in a room with a 12-hour light cycle (lights out at 6 pm) and allowed *ad libitum* access to standard NIH chow (NIH-31; LabDiet, Inc.) and water.

### Primary culture of hThyros

Cultures of hThyros were generated by isolating cells from normal thyroid tissue from patients undergoing thyroidectomy for thyroid tumors at the National Institutes of Health Clinical Center as previously described.^19^ The use of human samples was reviewed and approved by the NIDDK Institutional Review Board (Protocol number 77-DK-0096), and written informed consent was obtained from all participating patients.

Cells were maintained in DMEM with 10% FBS, 10 mM HEPES buffer (pH 7.4), 100 U/mL penicillin, and 100 μg/mL streptomycin (growth medium), and incubated at 37°C in a humidified atmosphere with 5% CO_2_. hThyros were utilized only between passages 3 and 5. For experiments, the cells were seeded in 48- or 12-well plates at a density of 3–4 × 10^4^ cells per cm^2^ in growth medium. The following day, cell growth was arrested overnight in serum-free medium containing the same components as the growth medium, except that FBS was replaced with 0.1% BSA (arresting medium).

### mRNA expression of thyroid genes in hThyros

hThyros were treated with either increasing doses of bTSH or 1 mU/mL bTSH with or without 10 µM Lins. After 5 days, the cells were lysed with RNA lysis buffer (RLT) buffer for RNA extraction. The levels of hTG and hTPO mRNAs were quantified from the total RNA using RNeasy Mini Kits, followed by reverse transcription to generate first-strand cDNA with the High-Capacity cDNA Archive Kit. Quantitative RT-PCR was then performed using the synthesized cDNA and iTaq™ Universal Probe Supermix. The results were normalized to human glyceraldehyde-3-phosphate dehydrogenase (hGAPDH).

### hTG and hTPO protein in hThyros

Secreted hTG was quantified in conditioned media after a 5-day treatment with bTSH (1 mU/mL) and Lins (10 µM) using an hTG ELISA kit, following the manufacturer’s instructions. Cell-associated hTG and hTPO were analyzed by Western blotting. Whole-cell lysates prepared with RIPA buffer were separated via SDS-polyacrylamide gel electrophoresis (PAGE) under reducing conditions on 4 − 12% gradient Precast NuPAGE gels using MOPS SDS running buffer, followed by transfer to nitrocellulose membranes using a Mini Trans-Blot® electrophoretic transfer cell (Bio-Rad). Membranes were blocked with Odyssey blocking buffer, and primary antibodies (TG 1:10,000; TPO and β-tubulin 1:1000) were diluted in Odyssey blocking buffer with 0.1% Tween® 20. The membranes were incubated overnight at 4°C with gentle shaking. After washing with TBS containing 0.1% Tween® 20, IRDye secondary antibodies (1:10,000) were added in the same buffer for 1 hour, followed by additional washes with TBS containing 0.1% Tween® 20 and tris-buffered saline (TBS) alone. The membranes were then imaged using an Odyssey CLx with the AutoScan function. Densitometric analysis was performed with Li-COR Image Studio^TM^ Version 2.1, and hTG and hTPO protein expression levels were normalized to total β-tubulin and the maximum TSH response.

### Pulsed stable isotope labeling using amino acids in cell culture (pSILAC)

hThyros were subjected to a 30-min starvation in SILAC arresting media lacking lysine and arginine with either dimethyl sulfoxide (DMSO) or 10 µM Lins pretreatment. Then, the pretreatment medium was replaced with arresting medium in DMEM for SILAC supplemented with labeled lysine and arginine under the following conditions: 1 mU/mL bTSH with isotopically medium amino acids; 1 mU/mL bTSH with 10 µM Lins with heavy amino acids; master control samples: 1 mU/mL bTSH with normal (light) amino acids. After 3 hours, monolayers were washed three times with ice cold PBS on ice, and cells were scraped with PBS. Wells with medium and heavy amino acids were combined in Diagenode tubes. Samples were centrifuged for 5 minutes at 1500 rpm at 4°C in Eppendorf Centrifuge 5425 R, PBS was aspirated, and cell pellets were frozen on dry ice.

hTG was analyzed by mass spectrometry. Briefly, cells were processed using soap lysis^20^ and thorough sonication. Lysates were reduced and alkylated (and excess alkylating agent scavenged) and then digested with trypsin. The soap was removed through acidification and ethyl acetate extraction. The same portion of an “L-only” sample was added to the “experiment” samples with thorough mixing before all samples were purified offline using reversed phase stage tips.^21^ Data-dependent analysis of liquid chromatography with tandem mass spectrometry (LC/MS/MS) experiments were performed on the “correction” and doped “experiment” samples and analyzed using MaxQuant.^22^ Corrections for extant “L” material in the “experiment” samples were made algebraically using protein level ratios, which are medians of relevant observations.^23^

### Lins effect on thyroid function *in vivo*

Female Wild-type BALB/c mice (3 experiments, total *n* = 96) were assigned to one of four experimental conditions (24 mice per group, across 3 experiments): (1) Lins vehicle (10% dimethylacetamide, 10% Kolliphor ELP, 80% H_2_O) once-daily for 3 days and a dose of 0.9% saline 1 hour after the last Lins injection; (2) Lins (15 mg/kg) once-daily for 3 days and a dose of 0.9% saline 1 hour after the last Lins injection; (3) Lins vehicle once-daily for 3 days and a dose of bTSH (1.0 mg/kg) 1 hour after the last Lins injection; (4) Lins (15 mg/kg) once-daily for 3 days and a dose of bTSH (1.0 mg/kg) 1 hour after the last Lins injection. All injections were given intraperitoneally (IP) in a volume of 10 ml/kg. Doses were calculated individually based on each mouse’s body weight; average mouse weight was 20 g. On the 3^rd^ day, 3 hours after bTSH/saline injection, a terminal blood sample was drawn, thyroids were extracted and flash frozen, and mice were euthanized by cervical dislocation under 5%-isoflurane anesthesia after their deep plane of anesthesia was confirmed by visual observation of respiratory rate as well as absence of pedal reflex.

### mRNA expression of thyroid genes in mouse thyroids

Following terminal retroorbital bleeding of anesthetized mice and euthanasia, thyroids were quickly dissected and flash-frozen in liquid nitrogen. For total RNA extraction, the tissue was homogenized in ice-cold RLT lysis buffer and purified using RNeasy Micro Kits. qRT-PCRs were performed as described above for hThyros with mouse-specific probes.

### Free thyroxine in mouse plasma

Following terminal retroorbital bleeding of anesthetized mice with ammonium heparinized Natelson collection tubes, samples were centrifuged at 6000 *g* for 15 minutes at RT. Plasma was transferred to another tube and kept at 4°C for measurement the next day. Free thyroxine (fT4) was measured using an ELISA kit according to manufacturer’s protocol with one modification: TMB reaction time was increased from 15 to 20 minutes. Raw absorbance values were corrected for optical aberration by subtracting 620 nm absorbance from the 450 nm TMB absorbance.

### Statistical analysis

All analyses were conducted using GraphPad Prism 10. For *in vivo* experiments, data were first examined for normality using D’Agostino–Pearson, Anderson–Darling, Shapiro–Wilk, and Kolmogorov–Smirnov tests as well as a visual inspection of a QQ plot. The fT4 data in all cases appeared to be normally distributed supporting the use of parametric testing. Statistical significance was assessed by two-way ANOVA and Holm-Šídák post-hoc tests to correct for multiple comparisons. Each mouse represented one fT4 data point for statistical analysis and all data points were included in the analysis. *p*-Values are reported in the figure legends. Two-tailed significance testing was conducted using a *p* value of 0.05 as the threshold for significance.

## Results

### Lins inhibits TSH-mediated upregulation of hTPO, but not of hTG mRNA, in hThyros

**FIG. 1.**
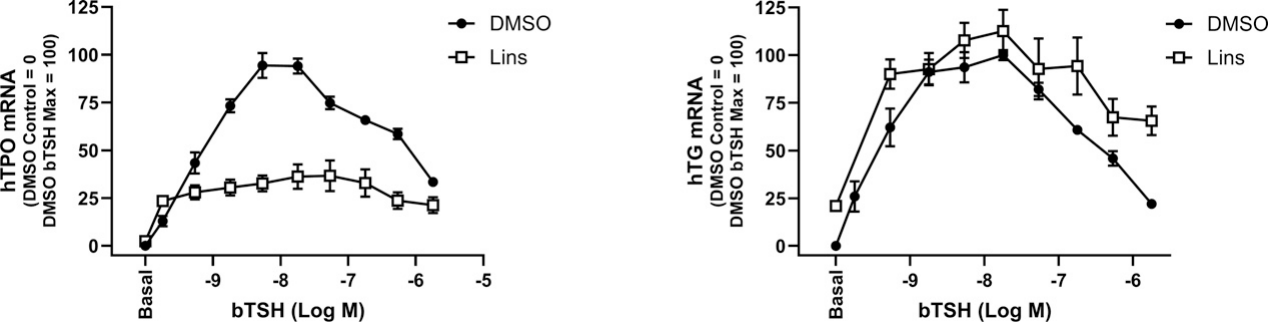
Lins inhibits bTSH-induced mRNA upregulation of hTPO but not of hTG in hThyros. hThyros were seeded at 1 × 10^5^ cells per well, allowed to attach overnight, then stimulated in arresting medium with 10 µM Lins alone or in combination with bTSH dose response. After 5 days of treatment, cells were lysed and analyzed for hTG, and hTPO mRNA levels using quantitative RT-PCR. Each experiment contained biological duplicates. Data are expressed as mean ± SEM; *n* = 3 patient donors. TSH, thyrotropin; TG, thyroglobulin; TPO, thyroperoxidase; h, human.

Previously, Lins was known to markedly inhibit TSH-mediated human sodium iodide symporter (hNIS) mRNA upregulation in a dose-dependent manner, whereas it had no effect on hTG mRNA levels in hThyros.^5^ Here, we studied the effect of Lins on hTG and hTPO mRNA and protein expression in more detail. We compared dose responses to TSH added alone or concomitantly with 10 µM Lins on hTG and hTPO mRNA (Fig. 1). Lins showed an inhibitory effect on the mRNA levels at every concentration of bTSH for hTPO. At 1 mU/mL bTSH (maximum bTSH effect), hTPO was inhibited by 61.5%. We confirmed the absence of the inhibitory Lins effect on hTG mRNA expression (Fig. 1).

### Lins inhibits TPO protein expression in hThyros

**FIG. 2.**
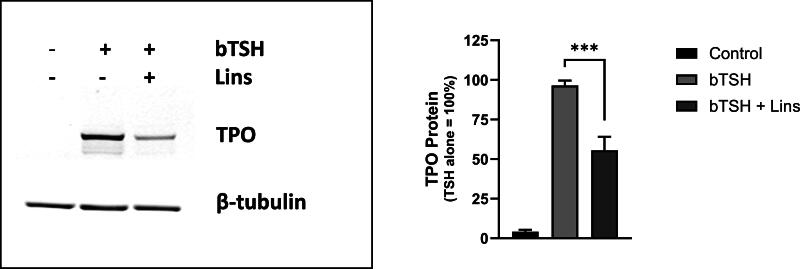
Lins inhibits bTSH-stimulated hTPO protein levels. hThyros were seeded at a density of 1 × 10^5^ cells per well, allowed to adhere overnight, and then stimulated in arresting medium, with 10 µM Lins alone or in combination with 1 mU/mL bTSH. After 5 days, cells were lysed, and hTPO expression was analyzed by Western blot. A representative blot from three independent experiments is shown **(A)**. Protein levels were quantified and normalized to β-tubulin **(B)**. Data are presented as the mean ± SEM, expressed as a percentage of the bTSH-only response; *n* = 3 patient donors. Group differences were assessed using an unpaired Student’s *t-test*, with a highly significant difference observed between the bTSH-treated and bTSH + Lins-treated wells (*p* = 0.0007). Lins, linsitinib.

We established that Lins is inhibitory of bTSH-induced hTPO mRNA levels (Fig. 1). We wanted to verify whether hTPO protein is also inhibited. Western blot demonstrated a reduction by 42.4% in the samples cotreated with 1 mU/mL bTSH and 10 µM Lins compared with bTSH alone (Fig. 2), confirming that Lins’ inhibition of hTPO mRNA correlates with the protein decreases.

### Lins has no effect on hTG mRNA but inhibits cell-associated hTG and its secretion in hThyros

**FIG. 3.**
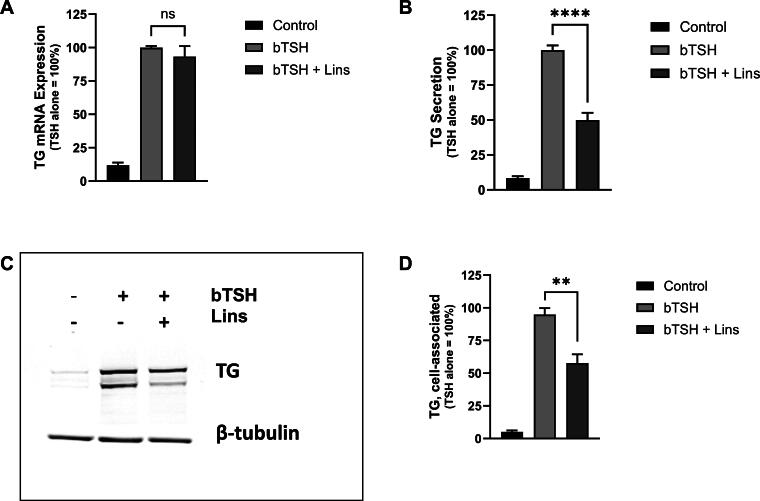
Lins decreases bTSH-induced cell-associated hTG protein and hTG secretion but has no effect on hTG mRNA. hThyros were plated at a density of 1 × 10^5^ cells per well, allowed to adhere overnight, and then stimulated in arresting medium, with 10 µM Lins either alone or in combination with 1 mU/mL bTSH. After 5 days, hTG mRNA expression was quantified using RT-PCR **(A)**, secreted hTG was measured in the conditioned media via ELISA **(B)**, and hTG expression in cell lysates was analyzed by Western blot **(C–D)**. A representative blot from three independent experiments is shown **(C)**. Protein expression levels were quantified and normalized to β-tubulin **(D)**. Data are presented as the mean ± SEM, expressed as a percentage of the bTSH-only response; *n* = 3 patient donors. Differences between groups were assessed using an unpaired Student’s *t-test*. Significant differences were observed between the bTSH-treated and bTSH + Lins-treated wells for hTG secretion (**B**, *p* < 0.0001) and cell-associated hTG (**D**, *p* = 0.0012), but not for mRNA expression **(A)**.

Due to Lins’ lack of impact on hTG mRNA (Fig. 1, 3A), we compared hTG mRNA levels with hTG protein levels. Combination treatment with 1 mU/mL bTSH and 10 µM Lins decreased cell-associated hTG protein by 39.1% and hTG secreted into the cell-cultured media by 50.1% (Fig. 3B–D). Thus, we established that the Lins-mediated decrease in hTG protein occurred in the absence of hTG mRNA decrease.

### Lins inhibits hTG translation in hThyros

**FIG. 4.**
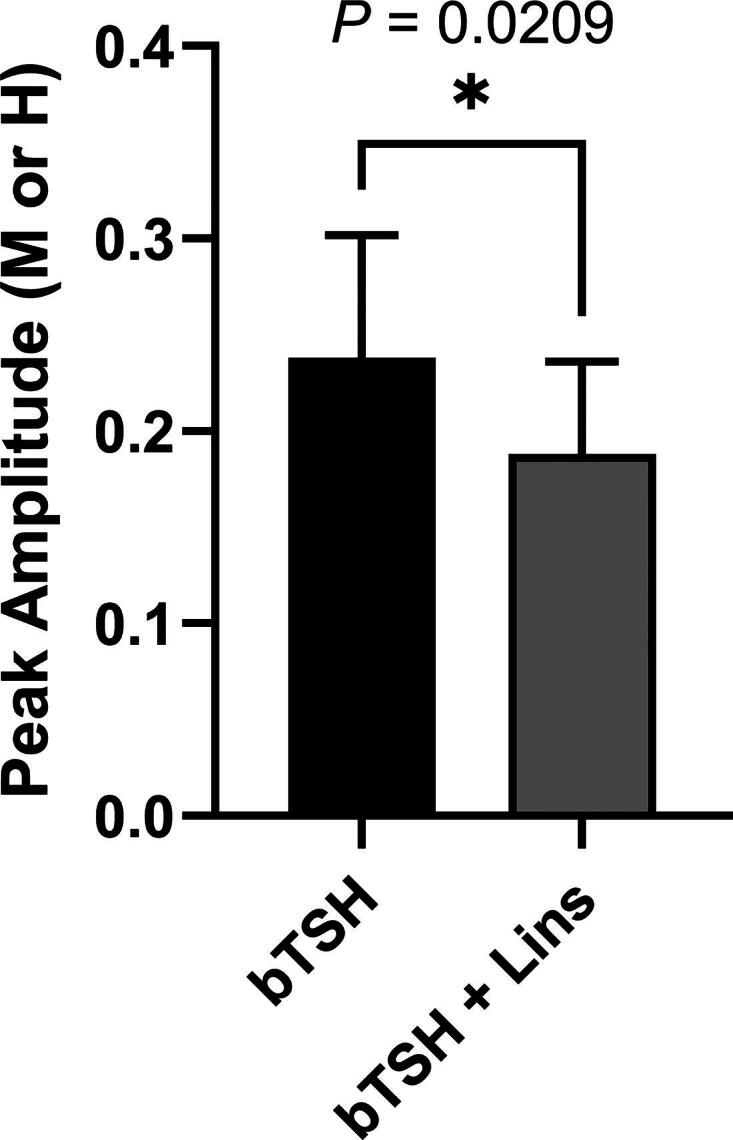
Lins effect on hTG translation measured by pSILAC. hThyros were seeded at 1 × 10^5^ cells/well in growth media in 12-well plates and allowed to attach overnight. Next day, growth media was replaced with arresting media for 24 hours. The following day, cells were subjected to a 30 minutes starvation in pSILAC arresting media lacking lysine and arginine with either DMSO or 10 µM Lins pretreatment. Then pretreatment medium was aspirated and replaced with arresting medium in DMEM for pSILAC supplemented with labeled amino acids (lysine and arginine) under the following conditions: 1 mU/mL bTSH with isotopically medium amino acids **(M)**; 1 mU/mL bTSH with 10 µM Lins with heavy amino acids **(H)** Arg and Lys; master control samples: 1 mU/mL bTSH with normal (light) amino acids. After 3 hours, monolayers were washed 3 times with ice cold PBS on ice and cells scraped with 200 µL PBS. Data are shown as mean ± SEM of the amplitudes of the spikes of the bTSH only and bTSH+Lins responses; *n* = 7 patient donors. The difference between groups was analyzed using paired Student’s *t-test*. The difference between wells treated with bTSH and bTSH + Lins was significant (*p* = 0.0209). PBS, phosphate-buffered saline.

Because cell-associated and secreted hTG were decreased in the absence of any change in hTG mRNA, we hypothesized that the decrease of hTG protein occurs at the level of translation. We performed pSILAC to compare the quantitative rate of hTG translation into protein in hThyros incubated with 1 mU/mL bTSH alone or a combination of bTSH and 10 µM Lins. After 3 hours of treatment, newly synthesized hTG peptides detected by mass spectroscopy in samples treated with Lins were 21% lower than in the samples treated with bTSH alone (Fig. 4), supporting our hypothesis that the decreases in hTG protein by Lins occurred at the level of translation.

### Lins inhibits mTpo mRNA *in vivo*

**FIG. 5.**
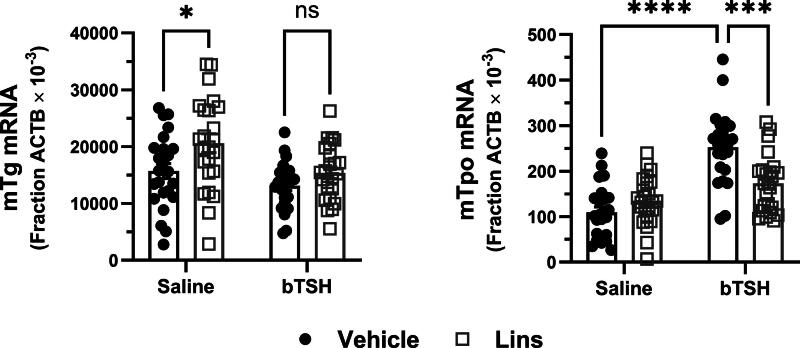
Lins inhibits TSH-stimulated m*Tpo* but not m*Tg* mRNA expression *in vivo.* Lins (15 mg/kg) was administered i.p. once daily on 3 consecutive days. On the third day, bTSH was administered i.p. 1 hour after the last dose of Lins, and 3 hours later the mice were sacrificed for sample collection. Thyroid genes were measured in mice treated with vehicle/saline, vehicle/bTSH, Lins/saline, and Lins/bTSH. The increase of m*Tpo* mRNA induced by bTSH was significantly inhibited with Lins treatment. In contrast, Lins had no effect on m*Tg* mRNA. Two-way ANOVA statistical analysis was used for significance testing. The data represent the summary of three independent experiments.

We confirmed the observed *in vitro* effects of Lins in mouse experiments. Lins (15 mg/kg) was administered i.p. once daily on three consecutive days. On the third day, bTSH was administered i.p. 1 hour after the last dose of Lins, and 3 hours later, the mice were sacrificed for sample collection. mRNA expression was measured in total RNA preparations from the mouse thyroid glands. In these experiments, bTSH had little or no effect on m*Tg* mRNAs but caused a significant increase in m*Tpo* mRNA. This exogenous bTSH-mediated increase in m*Tpo* mRNA was significantly inhibited by Lins compared with mice treated with bTSH only (Fig. 5).

### Lins decreases fT4 *in vivo*

**FIG. 6.**
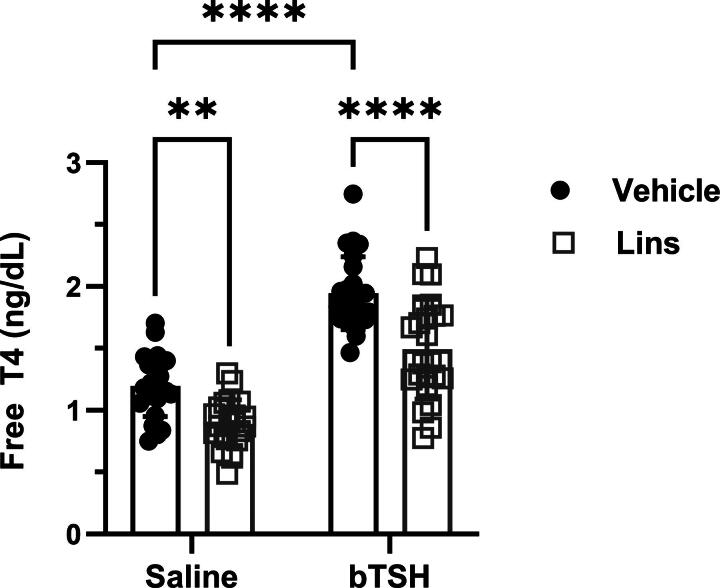
Lins decreases fT4 levels *in vivo.* Lins (15 mg/kg) was administered i.p. once daily on 3 consecutive days. On the third day, bTSH was administered i.p. 1 hour after the last dose of Lins, and 3 hours later the mice were sacrificed for sample collection. fT4 was measured in mice treated with vehicle/saline, vehicle/bTSH, Lins/saline, and Lins/bTSH. Basal fT4 was significantly lower in Lins-treated mice (*p* = 0.0013). bTSH caused a significant increase in fT4 overall (bTSH main effect *p* < 0.0001). The increase of fT4 induced by bTSH was significantly inhibited with Lins treatment (*p* < 0.0001). Two-way ANOVA statistical analysis was used for significance testing. The data represent the summary of three independent experiments. fT4, free thyroxine.

We measured fT4 plasma levels in mice subjected to Lins treatment under the experimental conditions described above. Lins decreased basal fT4 levels by 25.4%. Exogenous bTSH administration resulted in upregulation of fT4 over basal by 61.5%, which in turn was decreased by Lins by 23.2% (Fig. 6). Lins’ inhibitory effect on physiological responses in mice aligns with our observations in hThyros.

## Discussion

GD, the predominant etiology of hyperthyroidism, results from TSAbs persistently activating the TSHR, increasing thyroid hormone production.^24^ Previously, we found that TSH causes a biphasic cAMP response in hThyros, stimulating at low doses and inhibiting at high doses^25^ due to TSH receptor homodimerization, preventing hyperstimulation.^26,27^ However, only TSH, not TSAbs, caused this biphasic response in thyroid hormone biosynthetic gene expression,^28^ suggesting that high TSAb levels in GD lead to chronic hyperstimulation of thyrocytes.

New therapies for GD and TED redirected attention toward the IGF-1R, a crosstalk partner of TSHR that plays a significant role in their pathogenesis. Teprotumumab, an inhibitory IGF-1R antibody, is the only FDA-approved treatment for TED. To minimize adverse effects, teprotumumab infusion was administered once every three weeks for a total of eight infusions. Lins was previously studied in clinical trials as an experimental drug candidate for the treatment of various types of cancer. It was found safe and well tolerated.^29,30^ Lins, the IGF-1R small molecule antagonist, provides notable advantages over teprotumumab: it is orally available, making it easier to administer and potentially improving patient compliance, and it is more cost-effective, making it a more accessible treatment option. Therefore, if Lins proves to be effective in treating TED, it could potentially offer a more advantageous medical treatment for TED compared to teprotumumab.

We previously demonstrated in hThyros that Lins inhibited the upregulation of human NIS protein stimulated by TSH alone via the inhibition of crosstalk between TSHR and IGF-1R, without agonist activation of IGF-1R, via a mechanism involving extracellular signal-regulated kinase 1 and 2 (ERK1/2) and/or protein kinase B (Akt). Moreover, we showed that the crosstalk between TSHR and IGF1R in both GOFs and hThyros requires proximity of TSHR and IGF1R, with beta-arrestin 1 serving as a scaffold.^6^

Lins has been shown to inhibit TSHR/IGF-1R crosstalk in cells involved in TED pathogenesis (GOFs and fibrocytes)^15,16^ and in thyrocytes stimulated by TSH and TSAbs.^8^ A mouse model of GD was developed using plasmid-mediated close-field electroporation of the hTSHR A-subunit as an antigenic stimulus in female BALB/c mice.^31^ The immune response in this model leads to either hyperthyroidism or hypothyroidism, depending on the presence of stimulating or blocking TSHR antibodies, and progresses to include orbital inflammation.^32^ Recently, Lins has shown promise in preventing the autoimmune response in GD and TED in a mouse model^18^ and is currently in clinical trials for TED treatment (ClinicalTrials.gov Identifier: NCT05276063). However, it is important to recognize that TSHR/IGF-1R crosstalk is not limited to pathogenic autoimmune conditions; it also occurs in response to TSH stimulation in normal tissues. Thus, it is important to further investigate Lins’ effects on thyrocytes *in vitro* and its impact on thyroid function *in vivo*. For this study, we opted for TSH stimulation in wild-type female BALB/c mice as it ensures a consistent, robust, and well-timed increase in thyroid activity, which is less predictable in GD mice due to the gemish of stimulating and blocking TSHR antibodies. This approach allows for greater reproducibility in evaluating the effects of Lins in a physiological context.

We demonstrated that Lins inhibits bTSH-induced upregulation of hTPO at both mRNA and protein levels (Fig. 1 and 2). We confirmed our previous findings that Lins does not inhibit bTSH-induced upregulation of hTG mRNA^5^ (Fig. 1). However, we found that Lins decreased levels of both cell-associated and secreted hTG protein, suggesting that it inhibits the translation of TG mRNA. To test this hypothesis, we used pSILAC to measure the effect of Lins on the translation rate of hTG. As predicted, Lins inhibited the incorporation of heavy isotopes of Arg and Lys into newly synthesized hTG protein, decreasing amino acid incorporation by 21%. This is the first demonstration of Lins’ ability to inhibit translation in any cellular system. TG gene transcription in thyrocytes has been found to be highly and perpetually upregulated throughout the entire life of the cell in all major vertebrate groups.^33^ Given that TG transcription levels far exceed the expression of other thyroid-specific and house-keeping genes, it may remain elevated even when TSHR/IGF-1R crosstalk is inhibited by Lins in the presence of TSH. This compensatory mechanism to maintain homeostasis may explain the slight increase in TG mRNA observed in hThyros *in vitro* (Fig. 1) and mouse thyroids (Fig. 5) with Lins alone, despite the inhibition of TG protein and fT4 in both systems (Figs. 2, 6).

Our findings show that Lins effectively inhibits thyroid function in mice, which is an important consideration for its use in humans. We observed that Lins reduced basal and TSH-stimulated fT4 in mice. It is noteworthy to mention a discrepancy compared with Gulbins et al.^18^ who did not observe any effect of Lins on total T4 levels in their mice model. One possible explanation for this difference could be the concentration of Lins and the duration of its administration: we used 15 mg/kg for 3 days, whereas Gulbins et al. used 10 mg/kg for 4 weeks. Another plausible reason, not mutually exclusive, could be attributed to our measurement of fT4, which often offers greater sensitivity in detecting differences compared with total T4 (source: https://www.thyroid.org/thyroid-function-tests).

For future directions, it would be valuable to investigate the mechanisms underlying Lins’s effects on thyroid function in thyrocytes or GD mice at the transcriptome or proteome levels. These studies could offer deeper insights into the mechanisms of TSHR/IGF-1R crosstalk and the role of Lins in translation modulation.

In conclusion, our findings underscore the critical need to monitor thyroid function in patients relying on endogenous thyroid hormone production if Lins becomes available for TED treatment.
